# Upregulation of long noncoding RNA MIAT in aggressive form of chronic lymphocytic leukemias

**DOI:** 10.18632/oncotarget.11099

**Published:** 2016-08-05

**Authors:** Arash Sattari, Hasan Siddiqui, Farzaneh Moshiri, Apollinaire Ngankeu, Tatsuya Nakamura, Thomas J. Kipps, Carlo M. Croce

**Affiliations:** ^1^ Department of Molecular Virology, Immunology, and Medical Genetics and Comprehensive Cancer Center, The Ohio State University, Columbus, OH, USA; ^2^ School of Medicine and Surgery, Department of Public Health and Community Medicine, University of Verona, Verona, Italy; ^3^ Department of Medicine, Faculty of Medical sciences, Gorgan Branch, Islamic Azad University, Gorgan, Iran; ^4^ Department of Morphology, Experimental Medicine and Surgery, Section of Pathology, Oncology and Experimental Biology, University of Ferrara, Ferrara, Italy; ^5^ Department of Medicine, Moores Cancer Center, University of California at San Diego, La Jolla, CA, USA; ^6^ Chronic Lymphocytic Leukemia Research Consortium, San Diego, CA, USA; ^7^ Center for Childhood Cancer & Blood Diseases, The Research Institute at Nationwide Children's Hospital, Columbus, OH, USA

**Keywords:** long noncoding RNA MIAT, chronic lymphocytic leukemia, non-Hodgkin's lymphoma, OCT4, cell apoptosis

## Abstract

Long noncoding RNAs (lncRNAs) are non-proten-coding transcripts of more than 200 nucleotides generated by RNA polymerase II and their expressions are tightly regulated in cell type specific- and/or cellular differential stage specific- manner. MIAT, originally isolated as a candidate gene for myocardial infarction, encodes lncRNA (termed MIAT). Here, we determined the expression level of MIAT in established leukemia/lymphoma cell lines and found its upregulation in lymphoid but not in myeloid cell lineage with mature B cell phenotype. MIAT expression level was further determined in chronic lymphocytic leukemias (CLL), characterized by expansion of leukemic cells with mature B phenotype, to demonstrate relatively high occurrence of MIAT upregulation in aggressive form of CLL carrying either 17p-deletion, 11q-deletion, or Trisomy 12 over indolent form carrying 13p-deletion. Furthermore, we show that MIAT constitutes a regulatory loop with OCT4 in malignant mature B cell, as was previously reported in mouse pulripotent stem cell, and that both molecules are essential for cell survival.

## INTRODUCTION

Recently developed technology of next generation sequencing revealed that the majority of the human genome is transcribed, whereas only 1.5% to 2% of the transcripts encode proteins [1]. In addition, the degree of an organism complexity better correlates with the non-coding proportion of transcriptome than with the number of protein-coding genes [2]. Such findings imply an involvement of non-coding RNAs in eukaryotic evolution. Long non-coding RNAs (lncRNAs) are defined as RNA transcripts longer than 200 nucleotides in length. Similar to mRNAs, lncRNAs are transcribed by RNA polymerase II, are capped, contain introns and are often polyadenylated but they lack protein coding capacity [reviewed in ref. 3]. Known function of lncRNA includes i) scaffolds for chromatin modification complexes, ii) molecular guides to ensure the proper localization of their binding partners, iii) regulators for DNA looping, iv) interferer for RNA transcription, splicing, translation and stability, and v) co-activator for transcription factor [*ibid*]. In view of such multi-functional facets of lncRNAs, which encompass the fields of transcriptional and post-transcriptional regulation as well as subcellular dynamics, it is conceivable that dysregulation of lncRNA expression causes complex human diseases including human malignancies. Indeed, several lncRNAs have been shown to function as an oncogene or tumor suppressor [reviewed in ref.^4^]. MIAT/RNCR2, originally discovered as a candidate gene for myocardial infarction [5], is a lncRNA abundantly expressed in nervous system [6] and retinal tissue [7]. It has been shown that MIAT constitutes a unique nuclear structure by evading nuclear export. Because of its physical interaction with SF1 splicing factor, MIAT in the cell nucleus is supposed to be involved in RNA splicing and to exert a regulatory effect on gene expression [8]. In addition, Gomafu, the mouse homologue of MIAT, has been shown to bind to *Oct4* gene, which enhances Oct4 expression, and Oct4 binds to and positively regulates Gomafu transcription in mouse ES cells, and thus, they constitute a regulatory feedback loop [9]. Disruption of the regulatory loop by enforced suppression of Gomafu has been further shown to induce ES cell differentiation [*ibid.*]. These studies demonstrated a role of MIAT as a determinant for cell fate of neuronal, retinal, and embryonic stem cells [5, 7, 9]. At the moment, however, study of MIAT in hematopoietic cell lineage has not been reported yet. In the present study, we determined MIAT expression level quantitatively in leukemia/lymphoma cell lines, together covering a whole hematopoietic cell lineages to show MIAT up-regulation in lymphoid lineage with mature B phenotype including chronic lymphocytic leukemia (CLL) and non-Hodgkin's lymphoma. To further extend this finding, we determined MIAT expression level in primary CLL samples. This analysis showed that MIAT upregulation was frequently detected in aggressive forms of CLL defined by chromosomal abnormalities (the order of occurrence of MIAT upregulation: 17p del > 11q23 del = Trisomy 12) as compared to the MIAT expression level in 13p deletion. We further show that, as previously reported in mouse embryonic stem cells [9], MIAT in DLBL lymphoma cells constitutes a similar regulatory loop with OCT4 and in lymphoma, both MIAT and OCT4 are essential for cell survival. Upregulation of MIAT in aggressive forms of CLL and its biological activity in mature B cell malignancy indicate that MIAT may serve as a potential predictor for the disease outcome and its involvement in proliferation of the malignant B cells.

## RESULTS

### MIAT expression in leukemia/lymphoma cell lines and primary cells of CLL

**Figure 1:**
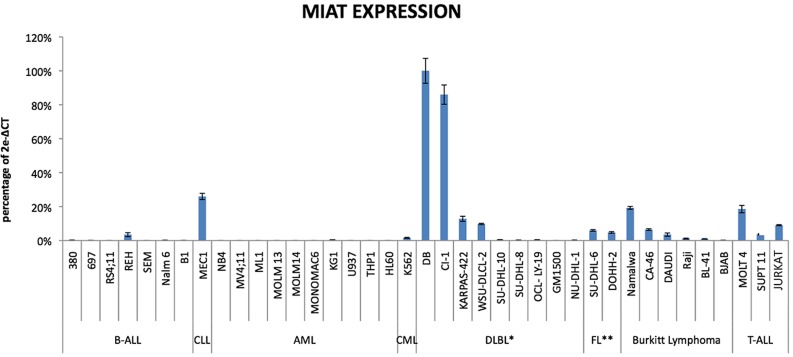
MIAT expression in Leukemia/Lymphoma cell lines MIAT expression was shown as percentage against expression level of TBP according to the following formula: 2e-(critical threshold of MIAT - critical threshold of TBP) × 100. Realtime RT-PCR was carried out in triplicate and standard error bar was on top. *: Diffuse Large B Cell Lymphoma. **: Follicular Lymphoma.

**Figure 2:**
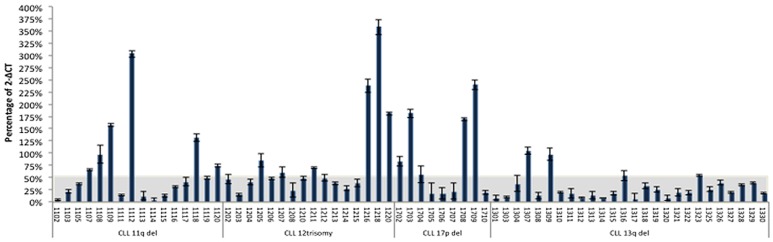
MIAT expression in primary leukemic cells from CLL patients MIAT expression was determined in patient samples, each having defined record of chromosome abnormality. Results were from triplicate assays. Gray colored area corresponds to the MIAT expression range of mean + standard deviation (50.8% +7.1%) calculated from 26 cases with 13q-deletion.

**Figure 3:**
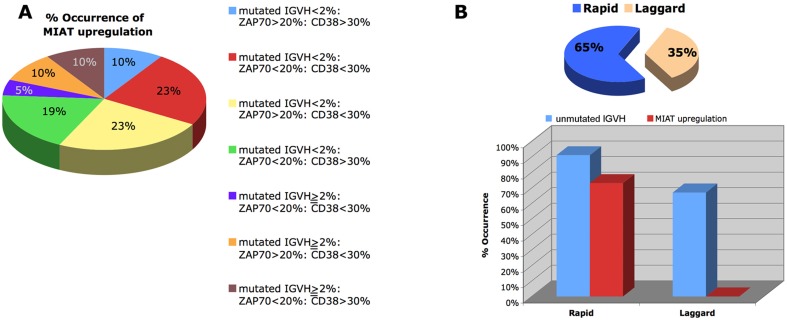
Inter-relation of MIAT upregulation with known prognostic markers in CLL **A.** Patients were divided into 7 subgroups based on the clinical laboratory data including percentage of CD38- or ZAP70-positive cells and percentage of mutated IGVH cells, and the frequency of each subgroup is shown as a pie chart. Percent occurrence of MIAT upregulation in each subgroup was shown in the chart. **B.** Dead cases were divided into rapid (within 6 years after diagnosis) and laggard (thereafter, 7 ~ 20 years after diagnosis) and their occurrence in percent were shown as a pie chart (top). Percent occurrences of MIAT upregulation and unmutated IGVH in each group were shown on the bottom.

We first determined MIAT expression level in 38 established leukemia/lymphoma cell lines including 7 B-cell acute lymphocytic leukemias with precursor B phenotype (BALL), one chronic lymphocytic leukemia (CLL), 10 acute myeloid leukemias (AML), one chronic myeloid leukemia (CML), 16 non-Hodgkin's lymphomas containing 8 diffuse large B cell lymphomas (DLBLs), 2 Follicular lymphomas (FL), and 6 Burkitt lymphomas, and 3 T-cell ALLs. Total RNAs obtained from these cell lines were subjected to cDNA synthesis, followed by quantitative real time PCR analysis using MIAT specific Taqman probe. In the present study, transcript of TATA binding protein (TBP), which is essential for execution of RNA polymerase II-mediated transcription including lncRNA production, were determined and used as an authentic normalizer to show MIAT expression level (Figure 1). This analysis showed overall low MIAT expression in B-ALL and myeloid lineage leukemic cells. In contrast, 4 out of 8 DLBLs, 2 out of 2 FLs and 3 out of 6 Burkitt cell lines were found to express MIAT at moderate to high level and among them, 2 DLBL cell lines, DB and CI-1 were shown to be high producer of MIAT. Notably, relatively high expression of MIAT, next to DB and CI-1 cells, was observed in a CLL cell line, MEC1. These results suggested that MIAT is expressed in malignant mature B cells but not in precursor B-ALL or in myeloid lineage leukemias. Based on this observation, we next determined MIAT expression in a total of 67 primary leukemic cells obtained from CLL patients, each having defined data of chromosomal abnormality specific for CLL (Figure 2). The course and outcome of CLL is highly heterogenous and CLLs are classified into either indolent or aggressive form [10]. Chromosome abnormality detected in CLL serves as a predictor to distinguish these 2 clinical forms. Thus, 13q deletion is frequently found in indolent form whereas 17p- or 11q-deletion frequently associates with aggressive form [11]. As shown in Figure 2, right most group carrying the 13q deletion showed low MIAT expression in average. This enabled to set up MIAT cut-off value as mean + standard deviation, calculated from 26 cases of this group (gray colored area in Figure 2). At this setting, percentage of MIAT upregulated cases in 13q del, 12 trisomy, 11q del, and 17p del were 15% (4/26 cases), 37% (6/16 cases), 37% (6/16 cases), and 55% (5/9 cases), respectively. When the two-tailed student's t-test was applied, MIAT expression in 11q del, 12 trisomy, and 17p del against the group carrying13p deletion was calculated by increase of 37% (p>0.014), 57% (p>0.002), and 61% (p>0.001), respectively. Next, we inspected the value of biomarkers known to be associated with disease aggressiveness including IGVH mutation, ZAP70-positive and CD38-positive percentage in MIAT upregulated 21 samples (Figure 3A). This analysis showed that MIAT upregulation coincided with unmutated IGVH (>98%) whereas ZAP70- (more than 20%) or CD38- (more than 30%) positive cases showed less linkage to MIAT upregulation. Studied patients in this article included 17 dead cases and 8 cases within this group showed MIAT upregulation (47%). This frequency is far less than that of unmutated IGVH (82%). However, when the death group was divided into rapid (death within 6 years after diagnosis, 11 cases) and laggard (death afterwards, 7 ~ 20 years, 6 cases), MIAT-upregulated cases were exclusively found in the former subgroup (Figure 3B). This made a great contrast to the occurrence of unmutated IGVH, which was found in both subgroups almost evenly [*ibid*]. Thus the results showed more close association of upregulated MIAT with rapid death cases.

### MIAT constitutes positive feedback regulatory loop with OCT4 and both molecules act on evading apoptotic cell death in malignant mature B cells

**Figure 4:**
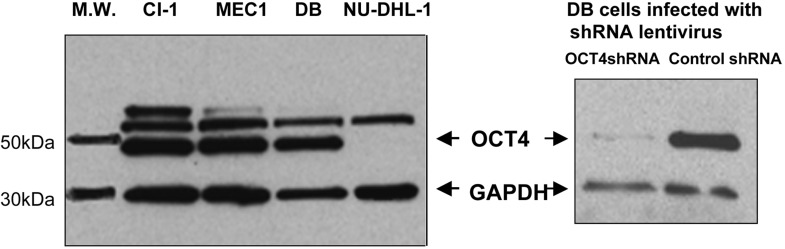
Western blot detection of OCT4 in leukemia/lymphoma cell lines expressing MIAT with different abundance 2 independent western blot results were shown as a composite figure. In each analysis, whole cell lysates were prepared from cells specified on top of the panel. On right panel, DB cells infected with lentivirus harboring OCT4shRNA or shRNA control (post-infection 72 hrs) were analyzed.

**Figure 5:**
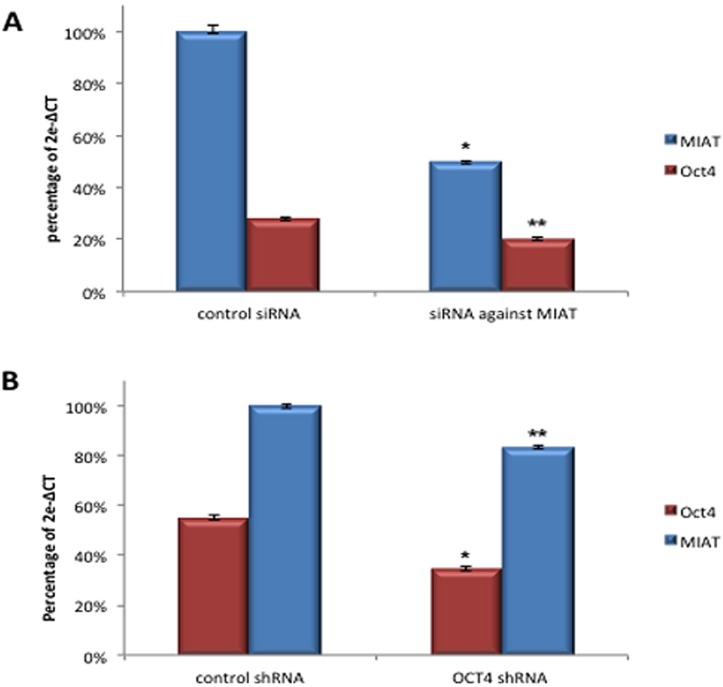
MIAT constitutes positive regulatory loop with OCT4 in transcription **A.** DB cells transfected with MIAT specific siRNA were analyzed by realtime RT-PCR for the quantitative detection of MIAT, OCT4, and TBP transcripts. Assay in biological triplicate was carried out post-transfection 24 hrs. *Reduced expression of the primary target (MIAT), *p* < 0.01. ** Concomitant downregulation of OCT4, *p* < 0.05. **B.** DB cells infected with OCT4shRNA lentivirus were analyzed by realtime RT-PCR. Assay in biological triplicate was carried out post-infection 72 hrs. * Reduced expression of the primary target (OCT4), *p* < 0.01. ** Concomitant downregulation of MIAT, *p* < 0.05. *p*-value was calculated by student t-test.

**Figure 6:**
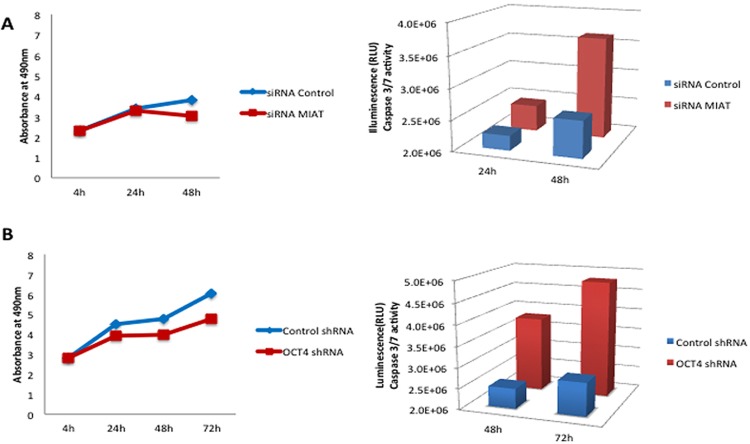
Enforced suppression of MIAT or OCT4 in DB cells induces cell apoptosis **A.** DB cells transfected with control siRNA or MIATsiRNA were assayed in biological triplicate for cell proliferation and Caspase 3/7 activities. **B.** DB cells infected with either control virus or OCT4shRNA harboring virus were assayed in biological triplicate for cell proliferation and Caspase 3/7 activities.

Current finding of MIAT upregulation in aggressive CLL prompted us to determine whether this lncRNA is directly involved in disease progression. To this end, we first investigated expression of OCT4, a sole known positive transcriptional regulator for MIAT at the moment, previously reported in mouse pluripotent stem cells [9]. Western blot detected 49kDa protein, which was profoundly suppressed by OCT4 knockdown (Figure 4, right panel) and therefore identified as OCT4. This analysis detected OCT4 in MEC1, CI-1 and DB cells but not in NU-DHL-1 cells (Figure 4, left panel). Since MIAT expression is moderate ~ high in the first 3 cell lines but not in the fourth line (see Figure 1), the western result indicated that MIAT in malignant mature B cells positively regulates OCT4 expression. To corroborate this possibility, we next suppressed MIAT by means of transfection with MIAT specific siRNA. For the following studies, we selected DB cells of DLBL origin, which exhibited highest efficiency for transfection as well as lentivirus infection among MIAT upregulated malignant B cell lines (data not shown). To accomplish effective MIAT knockdown, siRNA transfection was repeated 3 times at an interval of 24 hr. 24 hrs after the final transfection, manipulated cells were subjected to RT-PCR analysis for the quantitative detection of MIAT and OCT4 transcripts. Treatment with MIAT siRNA induced approx. 50% reduction of MIAT and concomitant downregulation of OCT4 (7.7% reduction, Figure 5A). This result indicated that MIAT in malignant B cells positively regulates OCT4 transcription as it does so in mouse ES cells. In addition, in the latter cells, it has been shown that OCT4 regulates MIAT expression and, thus, both molecules make up a positive feedback loop [9]. This possibility was further investigated by applying lentivirus-mediated transduction of OCT4 shRNA in DB cells. As shown in Figure 4 right panel and Figure 5B, western blot and real time RT-PCR analyses performed at post-infection 72 hrs showed effective suppression of OCT4 by transduction of OCT4 shRNA. Furthermore, we could detect concomitant downregulation of MIAT in OCT4 suppressed cells (16.3% reduction, Figure 5B). Collectively, these results showed that MIAT constitutes positive regulatory loop with its transcriptional regulator OCT4 and such molecular network is conserved between mouse pluripotent stem cells and human malignant mature B cells. During MIAT and OCT4 knockdown experiments, we noticed cell growth inhibition after suppression of MIAT or OCT4 as compared to the growth of control treated cells (Figure 6A and 6B, left panel). This indicated that loss of MIAT or OCT4 induces cell death of malignant mature B cells. To examine this possibility, we next assayed Caspase3/7, sensitive apoptotic markers, which enable to detect early changes towards cell death, in MIAT and OCT4 suppressed DB cells. This assay showed time-dependent increase of the caspase activity after MIAT suppression (Figure 6A, left). Fold increase against the activity determined in control treated cells at 24 and 48 hr after siMIAT transfection were 1.08 and 1.339, respectively. Similar result was also obtained from OCT4 suppressed cells, which showed time-dependant increase of the activity from 1.356-fold (at 48 hr) to 1.497-fold (at 72 hr) (Figure 6B, right). Finally, these results well reflected the inhibitory status in cell proliferation, shown by the growth curve and we concluded that MIAT and its transcriptional regulator OCT4 are required for cell survival of the malignant mature B cells.

## DISCUSSION

In the present study, we showed MIAT upregulation in malignant mature B cells including established cell lines of CLL and non-Hodgkin lymphoma origin as well as primary leukemic cells obtained from CLL patients. Importantly, in the latter, MIAT upregulation was shown to be associated with aggressive form of CLL, defined by chromosome abnormalities as well as clinical outcome. Further study of MIAT by using larger cohort would provide solid data to justify whether MIAT serves as a new biomarker for disease aggressiveness of chronic lymphocytic leukemias. In addition, due to the limitation of available clinical samples, the present study was conducted on peripheral blood mononuclear cell (PBMC) preparation. An issue is the presence of non-CLL cells in the preparation, which may affect the result of quantitative RT-PCR. However, remarkable lymphocytosis found in the studied cases suggests it less likely that contaminated non-CLL cells in our PBMC preparation undermine the present data of MIAT expression. Enrichment of CLL cell population by applying sorting with CD19 and CD5 markers would resolve this issue.

We showed that in malignant mature B cells, MIAT is regulated by OCT4, a sole known positive transcriptional regulator for MIAT initially discovered in mouse ES cells [9]. This result implied an involvement of OCT4 in MIAT upregulation found in malignant B cell lines and primary CLL cells. To support this notion, we found that OCT4 expression level in the established cell lines positively correlates with MIAT expression level (see Figure 4). To extend the result into primary CLL samples, we applied quantitative RT-PCR for the detection of OCT4 and compared its expression level with MIAT. In this study, we found that OCT4 expression level was low in average in MIAT low group, whereas it varied a lot in MIAT high group (data not shown). This result indicates that MIAT expression in CLL is not totally dependent on OCT4. Known factors, which lead to lncRNA dysregulation in cancer, include copy number alteration due to gene rearrangement such as deletion and amplification, cancer-linked single nucleotide polymorphism (SNP) occurred in lncRNA coding gene, epigenetic changes occurred in imprinted loci where lncRNA is embedded or changes of DNA methylation state occurred in promoter region of lncRNA, and transcriptional regulation via transcription factor such as oncoprotein MYC or tumor suppressor p53 [reviewed in ref.^4^]. We note that no cancer-associated copy number alteration or SNP has been reported at MIAT locus on chromosome 22q12.1.

We think it important in the present study that suppression of MIAT in malignant mature B cells resulted in cell apoptosis. For unknown reason, it is very difficult to obtain MIAT silencing with high efficiency by means of siRNA transfection. This was first recognized in ES cell study [9] and we also encountered similar difficulty. Among cell lines DB, CI-1 and MEC1, we could obtain approx. 50% reduction of MIAT after repetition of transfection (3 times) only in DB cells. This limited our study of investigating biological activity of MIAT to the non-Hodgkin's lymphoma cells. Although further study by applying different approach is needed to determine whether the anti-apoptotic function of MIAT is conserved in CLL, CLL cells are generally considered as low-grade non-Hodgkin lymphoma particularly in advanced stage. Therefore, we expect that the finding obtained from DB cells could be applicable to CLL cells. Upregulated MIAT may support monoclonal malignant B cell proliferation with naïve B-cell phenotype by evading cell apoptosis, and thus, contribute to disease progression. Similar function has been shown for CD38, a biomarker for disease progression of CLL [12]. CD38 is a trans-membrane molecule to drive CLL proliferation and chemotaxis via ZAP70-ERK1/2 signaling pathway [13]. Low linkage between MIAT expression level and CD38 or ZAP70 status (see Figure 3A) indicates that actual molecular events leading to protect CLL cells from apoptosis are different between them.

In summary, we showed that lncRNA MIAT is a potential new biomarker for aggressiveness of chronic lymphocytic leukemia and that MIAT functions to protect malignant mature B cells from cell apoptosis.

## MATERIALS AND METHODS

### Patients

CLL samples were obtained from patients enrolled in the CLL Research Consortium and on written informed consent in accordance with the Declaration of Helsinki. The study protocols were approved by the Institutional Review Boards of The Ohio State University. The participating institutions provided the clinical data associated with each patient at the time of sample collection. The samples were analyzed to determine expression of ZAP70, CD38, immunoglobulin heavy chain variable mutational status and karyotype, as previously described [10]. 67 peripheral blood mononuclear cells (PBMC) were isolated by density gradient centrifugation with Ficoll-PaquePlus (Amersham Biosciences, Uppsala, Sweden).

### Cell lines

A total of 38 leukemia/lymphoma cell lines were purchased either from the American Type Culture Collection (ATCC) or DSMZ (Deutsche Sammlung von Mikroorganismen und Zellkulturen, Germany). HEK-293T cells derived from human embryonic kidney and transformed with SV40 large T antigen were obtained from Invitrogen (Carlsbad, CA, USA). All leukemic cell lines were cultured in RPMI 1640 supplemented with10% FBS and Penicillin/streptomycin. DLBL cell line, OCI-LY-19 cells were cultured in α Minimal Essential Medium containing GlutaMAX™, supplemented with 20% FBS. HEK-293T cells were cultured in Dulbecco's Modified Eagle's Medium (DMEM) supplemented with 10% fetal bovine serum. Cells were maintained in a humidified incubator at 37°C and 5% CO2 and passaged every 2-3 days. Early passaged (passage 4-7) cell lines were used for transfection or lentivirus infection experiment.

### RNA extraction and real-time quantitative PCR

RNA from cultured cells and from patients with CLL was extracted with standard Trizol (Invitrogen) methods. Co-purified genomic DNA was removed by the treatment with DNase I (Thermo Fisher Scientific Inc., Amplification Grade, USA) according to the manufacturer's instructions. RNA extracted from lentivirus infected cells was further purified by using RNA Clean Up and Concentration Kit (Norgen Biotek, Canada). 80 ng of total RNA was subjected to cDNA synthesis by using Super Script VILO DNA synthesis Kit (Invitrogen, USA) according to the manufacturer's instructions. Real-time PCR analysis was carried out by using ABI PRISM 7900 Sequence Detection System. TaqMan^®^ Gene Expression Assay Kits for the detection of MIAT, OCT4 and TBP were Hs00402814, 4331182, and 433376, respectively (Life Technologies, USA). Assay was done in triplicates for each sample. The level of transcription was measured with Ct (threshold-cycle). The amount of target, normalized to an endogenous reference, is given by 2^−ΔCt^ (Comparative Ct method; Applied Biosystem).

### Gene silencing by siRNA transfection and lentivirus-mediated transduction of shRNA

For silencing MIAT, smart pool of 4 siRNAs directed against MIAT, designed to target all variants of MIAT (NCBI accession no. NR_033321, NR_033320, NR_003491, NR_033319) was purchased from Dharmacon. 100nM of MIAT siRNAs were transfected into DB cells (5×10^5^ cells in a well of 24 well plate) using Dharmafect transfection reagent (Dharmacon). For silencing OCT4, OCT4 shRNA Transfection Starter Kit (Dharmacon), composed of 4 shRNA constructs in pGIPZ vector and each targeting a unique region of *OCT4* gene, was purchased. The pGIPZ construct was cotransfected with lentiviral packaging mix (Thermo Scientific, Cat# TLP5912) into HEK-293T cells according to the manufacturer's instructions to generate lentivirus harboring shRNA against OCT4. Viral titer of approx. 1 × 10^6^ pfu/ml, titrated by counting GFP-transduced HEK293 cells (post-infection 48hr), could be obtained. Lentiviruses derived from 4 constructs were individually screened for their efficiencies of OCT4 suppression in HEK293T cells and the virus preparation from construct #41, which exhibited highest suppression was used for the subsequent studies. 1 × 10^5^ DB cells were infected with the lentivirus (2 × 10^5^ pfu, M.O.I.: 2) in a round-bottomed 5 ml polypropylene tube (Falcon), adjusted to 0.5ml with RPMI 1640 medium containing Polybrene (Sigma) at a concentration of 8μg/ml. Virus-host cell mixture in the tube was centrifuged at 2000 × g, for 3 hrs at RT, followed by an additional incubation for overnight at 37° (spin inoculation). Infected cells were washed, resuspended into 0.5ml of RPMI 1640 medium containing 10% FBS, and cultured for 48 ~ 72 hrs in a well of 24 well culture plate.

### Western blot analysis

Whole cell extracts were prepared from cells by adding RIPA lysis buffer (150mM NaCl, 0.1% SDS, 0.5% sodium deoxycholate, 1% NP-40) (Sigma) with complete protease inhibitor cocktails (Sigma). Cell lyzate was centrifuged at 13000 rpm for 15 min at 4°C and the supernatant was collected. Protein concentration was determined by using Bradford reagent (Bio-Rad). 30μg of protein was resolved in 4-15% Mini-PROTEAN TGX pre-cast gel (Bio-Rad), blotted onto a nitrocellulose membrane, and was probed with OCT4 antibody (Abcam ab19857) at a dilution of 1000. GAPDH detected on the same blot served as a loading control.

### Cell proliferation assay

Cell proliferation was determined by using The CellTiter 96^®^ AQueous One Solution kit (MTS) (Promega). 20μl of CellTiter 96^®^ AQueous One Solution Reagent was added into each well of the 96-well assay plate where 8 × 10^3^ cells in 100μl of culture medium were seeded. 1 hr after incubation at 37°C, 5% CO2, absorbance at 490nm was measured using a plate-reading illuminometer (infinite F200 PRO, Tecan Group). All of the experiments were performed in triplicate.

### Caspase-Glo 3/7 cell apoptosis assay

Caspase-3/7 activity was assayed by adding 100μl of Caspase-Glo^®^ 3/7 assay reagent (Promega) into each well of 96-well plate containing manipulated cells in 100μl media. Readings of blank well and control treated cells were served as assay control. Luminescence of each sample was measured by using plate-reading illuminometer (infinite F200 PRO, Tecan Group Ltd).

### Statistical analysis

Statistical significance was determined with two-tailed student's t-test by using GraphPad Prism 6.01. P-value of less than 0.05 was considered as significant. Confidence Level was at 95%.
